# Adjuvant radiation therapy in metastatic lymph nodes from melanoma

**DOI:** 10.1186/1748-717X-6-12

**Published:** 2011-02-06

**Authors:** Jean-Emmanuel Bibault, Sylvain Dewas, Xavier Mirabel, Laurent Mortier, Nicolas Penel, Luc Vanseymortier, Eric Lartigau

**Affiliations:** 1 Academic Radiotherapy Departement, CLCC Oscar Lambret Comprehensive Cancer Center, Lille-Nord de France University, LILLE, France; 2 Department of Dermatology, CHRU Lille, University Lille II, LILLE, France; 3 General Oncology Department, CLCC Oscar Lambret, University Lille II, LILLE, France

## Abstract

**Purpose:**

To analyze the outcome after adjuvant radiation therapy with standard fractionation regimen in metastatic lymph nodes (LN) from cutaneous melanoma.

**Patients and methods:**

86 successive patients (57 men) were treated for locally advanced melanoma in our institution. 60 patients (69%) underwent LN dissection followed by radiation therapy (RT), while 26 patients (31%) had no radiotherapy.

**Results:**

The median number of resected LN was 12 (1 to 36) with 2 metastases (1 to 28). Median survival after the first relapse was 31.8 months. Extracapsular extension was a significant prognostic factor for regional control (p = 0.019). Median total dose was 50 Gy (30 to 70 Gy). A standard fractionation regimen was used (2 Gy/fraction). Median number of fractions was 25 (10 to 44 fractions). Patients were treated with five fractions/week. Patients with extracapsular extension treated with surgery followed by RT (total dose ≥50 Gy) had a better regional control than patients treated by surgery followed by RT with a total dose <50 Gy (80% vs. 35% at 5-year follow-up; p = 0.004).

**Conclusion:**

Adjuvant radiotherapy was able to increase regional control in targeted sub-population (LN with extracapsular extension).

## Introduction

**Figure 1:**
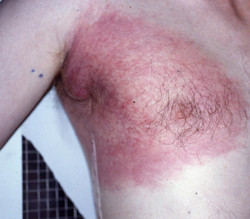
**Inflammatory axillary nodal recurrence from cutaneous melanoma**.

The incidence of cutaneous melanoma is increasing in fair-skinned populations. Surgery is the main treatment for melanoma and has a central role in the management of many patients [1]. Despite appropriate excision, locally invasive melanomas bring risks of both local and distant relapses [2]. While distant metastasis is often considered as the main factor for overall survival, regional control is still very important for the quality of life of these patients (figure 1). Systemic therapies for metastatic patients have led to modest improvements in locoregional control or overall survival [3]. Other ways to improve patients' survival have been explored in vain. The use of sentinel lymph node (SL) is gaining popularity in staging and treatment of patients with melanoma [4]. However, even with this approach, no survival benefit from SL with subsequent radical regional lymphadenectomy in malignant melanoma patients with lymph node (LN) metastases was found [5]. Additional treatments are therefore needed to improve the patient's outcome for melanomas with a high risk of locoregional or distant recurrence.

Radiation therapy forms the third cornerstone of cancer management, together with surgery and systemic treatments. Although the role of radiotherapy in achieving locoregional control and palliation is recognised, it is not often used for the management of melanoma. Use of radiation therapy for these patients has been hindered by the belief that melanoma is resistant to radiation [6]. This point of view is not shared by everyone [7].

Several retrospective studies on radiation therapy for the management of metastatic lymph nodes from cutaneous melanoma have been published [8-12]. They showed the benefit of radiation therapy in preventing local recurrence in metastatic lymph nodes from cutaneous melanoma after lymphadenectomy. This treatment had no impact on disease-free survival or overall survival. Most of these retrospective studies used a hypofractionated radiation regimen (30 Gy in 5 fractions).

In our centre, we chose to use a standard fractionation regimen for the management of these patients. In this study, we reviewed our experience in the treatment of locally advanced melanoma in order to identify prognostic factors. We tried to assess whether adjuvant radiation therapy was advantageous in locally advanced melanoma, which minimal dose and radiation regimen should be used, and for which patients it should be used.

## Material and methods

### Patients

Between 2000 and 2009, 86 successive patients were diagnosed with lymph node metastases from melanoma and treated with lymphadenectomy, followed by or without radiation therapy, and without systemic therapy.

Having four or more involved lymph nodes, extracapsular extension and node size greater than 3 centimetres were our main indications for radiation therapy in this study. Patients with visceral metastases at the time of RT were excluded from the analysis.

### Technical features of radiation therapy

Three-dimensional conformal radiation therapy was used. Areas treated included the axillary, cervical and groin lymph node areas. Organs at risk were contoured according to locations: for the axillary area: lung, heart, head of homolateral humerus; for cervical lymph nodes: parotid, larynx, thyroid; for groin lymph nodes: homolateral femoral head, rectum, bladder. Radiation was delivered by X-rays.

### Follow-up

Tumour relapse was established on the base of any clinical or radiological evidence of relapse. Any dermal, subcutaneous, soft tissue or lymph node relapse within or around the dissected and irradiated nodal basin was considered to be a local recurrence. The toxicity was analyzed using the grading scale introduced by Ballo *et al *in 2006 [9]. The classification consisted in grade 1 toxicity for an asymptomatic finding noted at the time of the follow-up physical examination; grade 2 for a symptomatic finding requiring any form of medical therapy (e.g., compressive sleeve for lymphedema, physical therapy for neuropathy, or long-term use of pain medication); and grade 3 for toxicity requiring surgical intervention. The follow-up period and survival were calculated from the date of surgery to November 2009.

### Statistical method

The distribution of categorical variables was tested using a Fisher exact test and chi-square test for trends. The primary endpoint was regional control, which was defined as complete and permanent eradication of tumour in treated area.. The secondary endpoint was overall survival. We carried out 3 successive analyses: (i) an identification of prognostic factors on the whole cohort (ii) a crude survival analysis according to the treatment performed (iii) a stratified survival analysis according to prognostic factor(s) identified.

Univariate analysis of the patients' survival was carried out using the Kaplan-Meier method with 95% confidence intervals (CI) and a log-rank comparison to evaluate the difference between the survival curves. Univariate analysis was performed according to Cox's proportional hazard. All statistical tests were two-sided, and a p value of <0.05 was considered statistically significant. The statistical package SPSS 13.0 (SPSS Inc., Chicago, IL, USA) was used to perform the analysis.

## Results

### Patient characteristics

Eighty-six patients were treated for metastatic lymph nodes from melanoma between August 1996 and November 2009. Fifty-seven were men. The median age at which the melanoma was diagnosed was 51 years (18 to 87 years). The median Breslow index was 2.5 mm (0.15 to 33 mm). The Clark level was known in 66 patients and was: level I in 1 patient, level II in 1 patient, level III in 16 patients, level IV in 44 patients and level V in 4 patients. Ulceration of the primary tumour was found in 12 patients. Initial treatment was not known for 2 patients. Seventy-nine patients had a complete resection of the initial melanoma (92%). Three patients had lymphadenectomy (3.4%) only, and 4 patients had concomitant resection of melanoma and lymphadenectomy (4.6%).

The clinical and pathologic characteristics are presented in Table 1. Median time lapse between initial diagnosis and lymph node metastases was 11 months (0 to 165 months). Median age when lymph node metastases were diagnosed was 52 years old (19 to 87 years old). The sites of the metastatic lymph nodes were: 20 cervical (23.3%), 26 axillary (30.2%) and 40 inguinal (46.5%). Twenty-six patients (30%) had no radiation therapy (group 1). Sixty patients (70%) underwent lymphadenectomy followed by conformal radiation therapy: 30 patients were treated with a total dose <50 Gy (group 2) and 30 patients with a total dose >50 Gy (group 3).

**Table 1:** Patients, tumors and lymph nodes characteristic according to treatments (surgery alone vs surgery followed by radiation therapy).

Characteristic	Surgery	Surgery + radiotherapy	
No of patients	26 (group 1)	60	

		Dose <50 Gy = 30 (group 2)	Dose >50 Gy = 30 (group 3)

Age (y)*	55 (27-87)	52 (18-87)	

Sex (F/M)	9/16	20/41	

Interval ME-NM (y)*	1(0-14)	1(0-12)	

**Metastatic LN site (No of patients)**			

Cervical	4	17	

Axillary	4	22	

Inguinal	18	21	

**Primary tumor**			

Unknown	0	1	

Breslow Index*	2.475 (0.38-33)	2.5 (0.15-33)	

Clark Level*	4 (4-3)	4 (1-5)	

Ulceration	3	9	

**Lymph node dissection**			

Number of resected LN*	11 (1-35)	11 (1-36)	

Number of positive LN*	1 (1-9)	2 (2-28)	

ECE*	1(0-3)	1 (0-11)	

Number of patients with LN size >3 cm	13	23	

* Median

Abbreviations: ME = Melanoma excision; NM = Node metastases; ECE = extracapsular extension; LN = lymph nodes

No systemic therapy was used for these patients until progression.

The median number of resected lymph nodes was 12 (1 to 36). The median number of positive lymph nodes was 2 (1 to 28). Forty-two patients presented at least one extracapsular extension (50.6%). The median total dose was 50 Gy (30 to 70 Gy). For a majority of treatments (37 patients; 63.8%) a standard fractionation regimen was used. The median dose/fraction was 2 Gy (1.8 to 3 Gy). The median number of fractions was 25 (10 to 44 fractions). Patients were treated with five fractions/week.

The median biological equivalent dose (BED with α/β = 2 Gy) was 50 Gy (18 to 71 Gy).

Median follow-up was 73 months (2 to 158 months). 18 patients were lost to follow-up.

#### Overall survival

Survival analysis was performed from the admitted date of lymph node recurrence. 43 patients (47.8%) died from an evolution of melanoma. Median survival after lymph node recurrence was 31.8 months ([CI] 23.3 to 40.3 months).

#### Regional control

16 patients (22.5%) presented a recurrence within the treated area.

### Prognostic factors

#### Regional control

Age (p = 0.2), sex (p = 0.64), initial site (p = 0.32), Breslow index (p = 0.88), Clark index (p = 0.7), number of resected lymph nodes (p = 0.2), number of metastatic lymph nodes (p = 0.88), and size of metastatic lymph node greater than 3 cm (p = 0.64) were not significantly associated with worse regional control. Extracapsular extension was significantly associated with worse regional control (p = 0.019).

#### Overall survival

**Figure 2:**
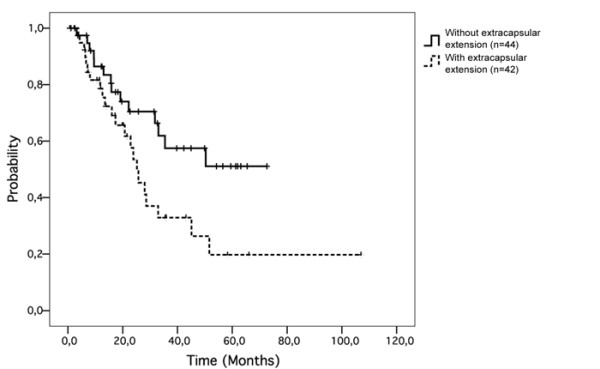
**Extracapsular extension was significantly associated with a worse overall survival (p = 0.03)**.

The following putative predictive factors were considered for the analysis: sex (p = 0.059), age (p = 0.3), time between initial cutaneous melanoma diagnosis and relapse (0.49), initial site (0.12), relapse site (0.25), Breslow index (0.7), Clark index (p = 0.1) and metastatic lymph node size greater than 3 cm (p = 0.2). Extracapsular extension was significantly associated with a worse survival (p = 0.03; figure 2).

### Outcome of patients with and without radiotherapy

**Figure 3:**
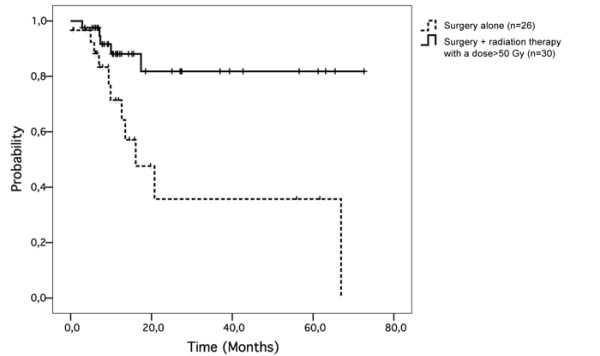
**Radiation therapy with a total dose of more than 50 Gy was associated with better regional control (p = 0.004)**.

**Figure 4:**
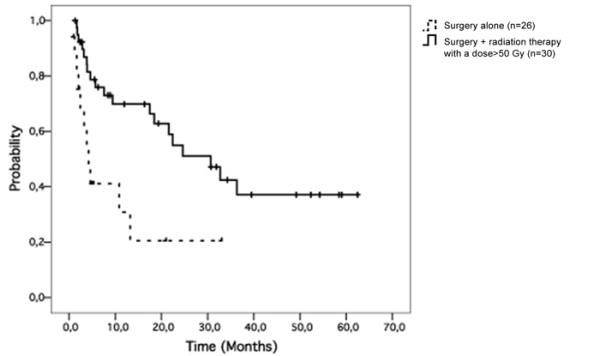
**A total dose of more than 50 Gy was associated with better overall survival (p = 0.005) for patients treated with surgery followed by radiation therapy**.

Radiation therapy did not improve regional control (p = 0.17) or overall survival (p = 0.18). Patients treated with a total dose >50 Gy (group 3) had better regional control (p = 0.004; figure 3) and overall survival (p = 0.005; figure 4) than patients treated by surgery alone (group 1).

Regional control rates for each tumour location were (radiation therapy >50 Gy vs. without radiation therapy): 90% vs. 70% for axillary LN metastasis, 80% vs. 72% for inguinal LN metastasis and 85% vs. 50% for cervical LN metastasis.

No statistical difference was found for regional control between the three LN metastasis locations (p = 0.4).

### Impact of radiotherapy after stratification according to the identified prognostic factor

**Figure 5:**
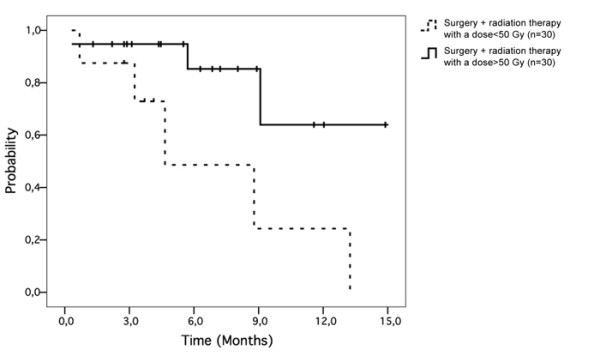
**Radiation therapy with a dose greater than 50 Gy was associated with better regional control (p = 0.03) for patients with extracapsular extension**.

An analysis stratified on extracapsular extension showed that patients with extracapsular extension treated with surgery followed by radiation therapy with a total dose ≥50 Gy (group 3) experienced a better regional control than those treated by surgery followed by adjuvant radiotherapy with a total dose <50 Gy (group 2): 80% vs. 35% at 5-year follow-up (p = 0.03; figure 5). This difference was not found for patients without extracapsular extension (p = 0.8).

### Toxicity

Grade 1 toxicity was found in 5 patients (5.5%). Grade 2 toxicity was found in 21 patients (23.3%): 6 patients (27.3%) treated with only surgery and 15 patients (22%) treated with surgery followed by radiation therapy. There was no grade 3 toxicity. Two patients (8.3%) treated for cervical LN metastasis, 4 patients (19%) treated for axillary LN metastasis and 15 patients (39.5%) for inguinal LN metastasis had grade 2 toxicity. Toxicity rates were 9% grade 1 and 9% grade 2 for cervical, 20% grade 2 for axillary and 45% for inguinal nodal regions.

There was a statistical increase in toxicity for patients treated for groin metastases (p = 0.01) compared to other treated areas, whichever treatment was performed (surgery alone or surgery followed by radiation therapy). No statistical correlation between radiation therapy and higher toxicity was found for cervical and inguinal regions (p > 0.05). There were more grade 2 toxicities for the axillary region when radiation therapy was used (p = 0.047). Dose >50 Gy was also not associated with higher toxicity (p = 0.36).

## Discussion

Our analysis aimed to identify the patient subgroups that could benefit from adjuvant radiation therapy. Extracapsular extension was the only significant prognostic factor for regional control and overall survival. Patients with this anatomopathologic feature were those who benefited the most from adjuvant radiation therapy. Our results are consistent with previous publications [13,14].

### Cervical nodal regions

In our study, regional control rate was 85% for cervical nodal metastasis for patients treated with surgery followed by radiotherapy. Recurrence rates for metastatic melanoma in cervical lymph nodes range from 30 to 50% after neck dissection alone [15-18]. Postoperative radiotherapy leads to regional control rates of about 90% for high-risk cervical metastases [19-21]. However, treatment-related morbidity is an issue with adjuvant RT. Toxicity was still very low for our patients: 9% grade 1 and 9% grade 2 toxicities. Ballo *et al *reported that 10% of patients had complications that required medical intervention at 5 years (ipsilateral hearing loss, hypothyroidism, wound breakdown and bone exposure) [10].

### Axillary nodal regions

Regional control rate in our study was 90% for patients treated with surgery and radiation therapy for axillary nodal regions. Axillary lymph node recurrence rates range from 23 to 50% [22,23]. Toxicity in our study was higher than that found for cervical nodal regions (20% grade 2 toxicity). Beadle *et al *reported treatment-related complications in 32% of patients treated with the hypofractionated regimen (30 Gy in five fractions, twice-weekly) after 5 years [11]. Lymphoedema occurred in 42 of 200 patients and was the most common complication. A study published by Starritt *et al *on lymphoedema occurrence in 107 patients treated with axillary dissection alone or axillary dissection plus postoperative radiation therapy [24] reported that lymphoedema occurred in 10% of patients who received dissection alone and in 53% treated with radiotherapy (p < 0.005). The hypofractionated regimen used in this study might explain this high rate.

### Inguinal nodal regions

The regional control rate for our patients treated with surgery and radiotherapy was 80%. However, the grade 2 toxicity rate was (45%). It is therefore important to select patients with high-risk anatomopathological features such as: extracapsular extension, 2 or more involved lymph nodes, or large nodal disease. Inguinal lymph node recurrence rates range from 19 to 40% of patients treated with dissection [17,25]. Ballo *et al *(8) reported a 3-year regional control of 74% in patients treated for high-risk inguinal nodal metastases with 30 Gy at 6 Gy per fraction. Complications are more common than in other tumour locations: 25 to 45% of patients were reported to develop lymphoedema [1,15,26-29]. Obesity (BMI >25 kg/m2) entailed higher rates of treatment-related complications (55%).

### Radiobiology of melanoma

Melanomas *in vitro *seem less radiosensitive than other tumour cell lines, but actually have a wide range of sensitivities [30-32]. Overgaard analyzed the radiation response of a clinical series of more than 600 metastatic melanoma lesions, mainly skin metastases [33]. One of the conclusions was that the response rate was dependent on the size of the fraction, with complete response rates of 57% when fractions of more than 4 Gy were used. This has led many to advocate a hypofractionated radiation therapy regimen. However, RTOG 83-05, the only study designed to assess whether a high dose per fraction irradiation was preferable in melanoma treatment, showed no difference in regional control between conventional and hypofractionated schedules [34]. A more recent study published in 2009 by Strojan *et al *[12] opted for more conventionally fractionated radiotherapy schedules (2-2.5 Gy/fractions).

Our study would also lead us to believe that a more conventionally fractionated schedule could be used (1.8 to 2.5 Gy) with a higher total dose (>50 Gy) in order to minimize toxicities. However, its unicentric, retrospective design and the limited number of patients included limits the interpretation of our results (our low toxicity rates might be underevaluated), even if our groups of patients were well balanced.

Overall, we would recommend using adjuvant radiation therapy for patients with lymph node metastases from cutaneous melanoma, especially if they present one or more LN with extracapsular extension. Total dose should be strictly greater than 50 Gy for this kind of treatment, with a standard fractionation regimen (ex: 2 Gy/fraction, 5 fractions a week) in order to maximize the efficacy and to minimize the toxicity. An even better approach would be to use the biological equivalent dose (BED), which should be greater than 50 Gy.

## Conclusion

Melanoma is often considered to be a radioresistant tumour. Our data, in accordance with previously reported series, show that adjuvant radiation therapy provides good regional control. However, since toxicity is not negligible, especially for axillary and inguinal lymph nodes, this treatment should be considered only for patients with poor anatomopathological features. The first factor that should be taken into account is extracapsular extension.

## Conflict of interest notification

The authors declare that they have no competing interests.

## Authors' contributions

JEB and XM conceived the study. JEB collected data and drafted the manuscript. SD, XM, LM, NP, LV and EL participated in coordination and helped to draft the manuscript. SD performed the statistical analyses. EL provided mentorship and edited the manuscript. All authors have read and approved the final manuscript.

## References

[B1] Hughes TMD Ahern RP Thomas JM Prognosis and surgical management of patients with palpable inguinal lymph node metastases from melanoma Br J Surg 2000 87 7 892 901 10.1046/j.1365-2168.2000.01439.x 10931025

[B2] Tsao H Atkins MB Sober AJ Management of cutaneous melanoma New England Journal of Medicine 2004 351 10 998 10.1056/NEJMra041245 15342808

[B3] Lens MB Dawes M Interferon Alfa Therapy for Malignant Melanoma: A Systematic Review of Randomized Controlled Trials J Clin Oncol 2002 20 7 1818 1825 10.1200/JCO.2002.07.070 11919239

[B4] Messina JL Glass LF Cruse CW Berman C Ku NK Reintgen DS Pathologic examination of the sentinel lymph node in malignant melanoma Am J Surg Path 1999 23 6 686 10.1097/00000478-199906000-00008 10366151

[B5] Rutkowski P Nowecki ZI Nasierowska-Guttmejer A Ruka W Lymph node status and survival in cutaneous malignant melanoma--sentinel lymph node biopsy impact Eur J Surg Onc 2003 29 7 611 618 10.1016/S0748-7983(03)00118-5 12943629

[B6] Barranco SC Romsdahl MM Humphrey RM The radiation response of human malignant melanoma cells grown in vitro Cancer Research 1971 31 6 830 5088486

[B7] Stevens G McKay MJ Dispelling the myths surrounding radiotherapy for treatment of cutaneous melanoma The Lancet Oncology 2006 7 7 575 583 10.1016/S1470-2045(06)70758-6 16814209

[B8] Ballo MT Zagars GK Gershenwald JE A critical assessment of adjuvant radiotherapy for inguinal lymph node metastases from melanoma Ann of Surg Onc 2004 11 12 1079 1084 10.1245/ASO.2004.12.039 15576833

[B9] Ballo MT Ross MI Cormier JN Combined-modality therapy for patients with regional nodal metastases from melanoma Int J Radiat Oncol Biol Phys 2006 64 1 106 113 10.1016/j.ijrobp.2005.06.030 16182463

[B10] Ballo MT Bonnen MD Garden AS Adjuvant irradiation for cervical lymph node metastases from melanoma Cancer 2003 97 7 1789 1796 10.1002/cncr.11243 12655537

[B11] Beadle BM Guadagnolo BA Ballo MT Radiation therapy field extent for adjuvant treatment of axillary metastases from malignant melanoma Int J Radiat Oncol Biol Phys 2009 73 5 1376 1382 10.1016/j.ijrobp.2008.06.1910 18774657

[B12] Strojan P Jancar B Cemazar M Perme MP Hocevar M Melanoma Metastases to the Neck Nodes: Role of Adjuvant Irradiation Int J Radiat Oncol Biol Phys 2010 77 4 1039 1045 10.1016/j.ijrobp.2009.06.071 19910139

[B13] Coit DG Rogatko A Brennan MF Prognostic factors in patients with melanoma metastatic to axillary or inguinal lymph nodes A multivariate analysis Ann Surg 1991 214 5 627 636 10.1097/00000658-199111000-00014 PMC1358620 1953117

[B14] Bevilacqua RG Coit DG Rogatko A Younes RN Brennan MF Axillary dissection in melanoma. Prognostic variables in node-positive patients Ann Surg 1990 212 2 125 131 10.1097/00000658-199008000-00002 2375645 PMC1358045

[B15] Bowsher WG Taylor BA Hughes LE Morbidity, mortality and local recurrence following regional node dissection for melanoma Br J Surg 1986 73 11 906 908 10.1002/bjs.1800731119 3790922

[B16] Byers RM The role of modified neck dissection in the treatment of cutaneous melanoma of the head and neck Arc of Surg 1986 121 11 1338 10.1001/archsurg.121.11.1338 3778208

[B17] Lee RJ Gibbs JF Proulx GM Nodal basin recurrence following lymph node dissection for melanoma: implications for adjuvant radiotherapy Int J Radiat Oncol Biol Phys 2000 46 2 467 474 10.1016/S0360-3016(99)00431-9 10661355

[B18] O'brien CJ Coates AS Petersen-Schaefer K Experience with 998 cutaneous melanomas of the head and neck over 30 years Am J Surg 1991 162 4 310 1951880 10.1016/0002-9610(91)90138-4

[B19] Shen P Wanek LA Morton DL Is adjuvant radiotherapy necessary after positive lymph node dissection in head and neck melanomas? Ann of Surg Onc 2000 7 8 554 559 10.1007/BF02725332 11005552

[B20] Calabro A Singletary SE Balch CM Patterns of relapse in 1001 consecutive patients with melanoma nodal metastases Arc of Surg 1989 124 9 1051 10.1001/archsurg.1989.01410090061014 2774907

[B21] Moncrieff MD Martin R O'Brien CJ Adjuvant Postoperative Radiotherapy to the Cervical Lymph Nodes in Cutaneous Melanoma: Is There Any Benefit for High-Risk Patients? Ann of Surg Onc 2008 15 11 3022 3027 10.1245/s10434-008-0087-8 18958539

[B22] Pidhorecky I Lee RJ Proulx G Risk factors for nodal recurrence after lymphadenectomy for melanoma Ann of Surg Onc 2001 8 2 109 115 10.1007/s10434-001-0109-2 11258774

[B23] Karakousis CP Hena MA Emrich LJ Axillary node dissection in malignant melanoma: results and complications Surgery 1990 108 1 10 2360176

[B24] Starritt EC Joseph D McKinnon JG Lymphedema after complete axillary node dissection for melanoma: assessment using a new, objective definition Ann Surg 2004 240 5 866 10.1097/01.sla.0000143271.32568.2b 15492570 PMC1356494

[B25] Gadd MA Coit DG Recurrence patterns and outcome in 1019 patients undergoing axillary or inguinal lymphadenectomy for melanoma Arc of Surg 1992 127 12 1412 10.1001/archsurg.1992.01420120046008 1365686

[B26] Allan CP Hayes AJ Thomas JM Ilioinguinal lymph node dissection for palpable metastatic melanoma to the groin J Surg 2008 78 11 982 10.1111/j.1445-2197.2008.04716.x 18959697

[B27] Karakousis CP Driscoll DL Rose B Groin dissection in malignant melanoma Ann of Surg Onc 1994 1 4 271 277 10.1007/BF02303564 7850524

[B28] Strobbe LJ Jonk A Hart AA Positive iliac and obturator nodes in melanoma: survival and prognostic factors Ann of Surg Onc 1999 6 3 255 262 10.1007/s10434-999-0255-5 10340884

[B29] Urist MM Maddox WA Kennedy JE Patient risk factors and surgical morbidity after regional lymphadenectomy in 204 melanoma patients Can J Clin 2006 51 11 2152 2156 10.1002/1097-0142(19830601)51:11<2152::aid-cncr2820511134>3.0.co;2-7 6839303

[B30] Rofstad EK Wahl A Brustad T Radiation sensitivity in vitro of cells isolated from human tumor surgical specimens Can Res 1987 47 1 106 3791195

[B31] Bentzen SM Overgaard J Thames HD Clinical radiobiology of malignant melanoma Radiotherapy and oncology: journal of the European Society for Therapeutic Radiology and Oncology 1989 16 3 169 2587808 10.1016/0167-8140(89)90017-0

[B32] McKay MJ Kefford RF The spectrum of in vitro radiosensitivity in four human melanoma cell lines is not accounted for by differential induction or rejoining of DNA double strand breaks Int J Radiat Oncol Biol Phys 1995 31 345 345 10.1016/0360-3016(94)E0147-C 7836088

[B33] Overgaard J Overgaard M Hansen PV Some factors of importance in the radiation treatment of malignant melanoma Radiother Oncol 1986 5 3 183 10.1016/S0167-8140(86)80048-2 3085169

[B34] Sause WT Cooper JS Rush S Fraction size in external beam radiation therapy in the treatment of melanoma Int J Radiat Oncol Biol Phys 1991 20 3 429 10.1016/0360-3016(91)90053-7 1995527

